# Mechanisms of airway epithelial injury and abnormal repair in asthma and COPD

**DOI:** 10.3389/fimmu.2023.1201658

**Published:** 2023-07-13

**Authors:** Katie Louise Raby, Charalambos Michaeloudes, James Tonkin, Kian Fan Chung, Pankaj Kumar Bhavsar

**Affiliations:** ^1^ National Heart and Lung Institute, Imperial College London, London, United Kingdom; ^2^ School of Medicine, European University Cyprus, Nicosia, Cyprus; ^3^ Department of Respiratory Medicine, Royal Brompton and Harefield Hospital, London, United Kingdom

**Keywords:** epithelium, injury, repair, asthma, COPD, barrier, cell junctions, permeability

## Abstract

The airway epithelium comprises of different cell types and acts as a physical barrier preventing pathogens, including inhaled particles and microbes, from entering the lungs. Goblet cells and submucosal glands produce mucus that traps pathogens, which are expelled from the respiratory tract by ciliated cells. Basal cells act as progenitor cells, differentiating into different epithelial cell types, to maintain homeostasis following injury. Adherens and tight junctions between cells maintain the epithelial barrier function and regulate the movement of molecules across it. In this review we discuss how abnormal epithelial structure and function, caused by chronic injury and abnormal repair, drives airway disease and specifically asthma and chronic obstructive pulmonary disease (COPD). In both diseases, inhaled allergens, pollutants and microbes disrupt junctional complexes and promote cell death, impairing the barrier function and leading to increased penetration of pathogens and a constant airway immune response. In asthma, the inflammatory response precipitates the epithelial injury and drives abnormal basal cell differentiation. This leads to reduced ciliated cells, goblet cell hyperplasia and increased epithelial mesenchymal transition, which contribute to impaired mucociliary clearance and airway remodelling. In COPD, chronic oxidative stress and inflammation trigger premature epithelial cell senescence, which contributes to loss of epithelial integrity and airway inflammation and remodelling. Increased numbers of basal cells showing deregulated differentiation, contributes to ciliary dysfunction and mucous hyperproduction in COPD airways. Defective antioxidant, antiviral and damage repair mechanisms, possibly due to genetic or epigenetic factors, may confer susceptibility to airway epithelial dysfunction in these diseases. The current evidence suggests that a constant cycle of injury and abnormal repair of the epithelium drives chronic airway inflammation and remodelling in asthma and COPD. Mechanistic understanding of injury susceptibility and damage response may lead to improved therapies for these diseases.

## 1 Introduction

The airway epithelium is a complex system of different cell types that works in conjunction with the immune system to form an intact barrier, which prevents foreign particles and pathogens from entering the submucosal compartment. The whole system works together as the first-line defence to protect and limit the damage caused by stress (1, 2). The mucociliary system traps and expels foreign particles from the respiratory tract. Furthermore, tight junctions and adherens junctions form cell to cell contacts, and regulate the flow of molecules across the basement membrane to the underlying sub-epithelium. However, defects in these intertwined processes and loss of barrier function can cause airway diseases, including chronic obstructive pulmonary disease (COPD) and asthma.

Chronic exposure to cigarette smoke, which is the main risk factor for COPD, as well as repeated infections, cause changes in the epithelium including loss of ciliated epithelial cells (3–6), increased goblet cell number (7), shortened cilia and reduced beat frequency (8, 9). Epithelial injury triggers a chronic immune response that drives alveolar destruction (emphysema), and airway remodelling that involves peribronchial fibrosis and possibly increased airway smooth muscle mass (1–5). Furthermore, the alterations in the cell type proportions, causes increased mucus production, a predominant feature of chronic bronchitis associated with COPD (10).

Severe asthma is a chronic inflammatory disease classified as heterogenous in relation to clinical phenotype, severity, and the response of the epithelium to injury. Epithelial injury in severe asthma, whether due to allergen, pollution, viral or bacterial insult, causes several changes in the response and integrity of the epithelium, leading to immune cell recruitment, inflammation, epithelial shedding (2, 11, 12). The asthmatic airway epithelial layer is disrupted as evidenced by the detachment of ciliated epithelial cells, and loss of cell-cell contacts that leads to reduced integrity and increased permeability (13–15). This allows for the release of pro-inflammatory cytokines, such as the alarmins IL-33 and IL-25 (16), and the Th2 cytokines IL-4 and IL-13 (14, 17). These mediators activate and recruit immune cells, which further damage the epithelial layer and contribute to patient exacerbations. Mediators produced by the injured epithelium and the local immune cells drive airway remodelling, which involves subepithelial fibrosis due to increased ECM deposition, and airway smooth muscle thickening caused by smooth muscle cell hyperplasia and/or hypertrophy (1).

In this review we will demonstrate how the structure and composition of the epithelium, and the basement membrane, and their interaction with the innate immune system facilitates homeostasis and protection of the airway. We will then discuss how structural and functional changes in the COPD and asthmatic epithelium further drive disease, exacerbations, and lung function decline (**Table 1**). Specifically, epithelial dysfunction in COPD is largely due to altered cell type composition and reduced mucociliary clearance in response to cigarette smoke exposure. On the other hand, in asthma, the reduced integrity and increased permeability is caused by loss of epithelial cell-cell contact and cell shedding due to chronic inflammation.

**Table 1:** Comparison of Asthma and COPD risk factors, pathogenesis and airway dysfunction manifestations.

	Asthma	COPD
**Clinical manifestations**	Variable respiratory symptoms; reversible expiratory airflow limitation associated with airway hyperresponsiveness. Cough and mucus production are common symptoms of asthma that are correlated with worse outcomes.	Chronic irreversible airflow obstruction, which manifests as shortness of breath, cough, and sputum production. Chronic bronchitis is also feature of COPD.
**Causes of epithelial dysfunction**	Reduced integrity and increased permeability due to loss of cell-cell contact, epithelial shedding caused by chronic inflammation.	Altered cell type composition and reduced mucociliary clearance in response to cigarette smoke
**Epithelial structure changes**	Increased basal cell number with reduced differentiation and proliferation capacity, Goblet cell hyperplasia.	Increased basal cell number with reduced differentiation and regeneration capacity; Squamous cell metaplasia; Goblet cell hyperplasia.
**Abnormal cell differentiation**	Increased EGFR: IL-13 induced inhibition of ciliated cell differentiation and increased differentiation of goblet cells; Downregulation of ciliated cell markers, reduced FoxJ1 and increased JAK/STAT and Notch signalling.	Increased EGFR in basal cells in response to cigarette smoke. EGF associated with increased goblet and squamous cell metaplasia. Increased goblet cells leading to increased mucus production.
**Changes in Cilia**	Dyskinetic cilia, reduced ciliary beat frequency.	Loss of cilia, shortened cilia length, reduced cilia beat frequency.
**Epithelial cell response to injury**	Ciliated cell detachment, increased immune cell recruitment, epithelial junction disassembly. Alarmin release through PRR and DAMP activation inducing Th2 response.	Increased ROS in response to cigarette smoke, Th1 immune cell recruitment through epithelial alarmin release. Increased neutrophil and macrophage infiltration.
**Oxidative stress**	Increased apoptosis of ciliated epithelial cells due to oxidative stress of mitochondria. Reduction in the epithelial barrier integrity and permeability due to cleaving of tight junction proteins. Reduced epithelial pore-forming claudins.	Autophagy in response to oxidative stress is reduced causing premature cell senescence, ROS dependent apoptosis and necroptosis of ciliated epithelial cells. Abnormal phosphorylation and redistribution of tight and adherent junctions.
**Inflammatory response**	Release of alarmins IL-25, TSLP and IL-33 from epithelial cells. Recruitment of Th2 immune cells, eosinophils, mast cells release IL-13, IL-4 and IL-5 inducing epithelial damage.	Epithelial cell release of pro-inflammatory cytokines CXCL8, G-CSF, LTB4 and MCP-1. Recruitment of monocytes, macrophages, and neutrophils. Release of IL-6 and TNF.

## 2 Epithelial structure and cell types

The airway epithelium is a continuous sheet of epithelial cells, comprised of different cell types: predominantly ciliated, goblet, basal and club cells. These cells form a strong attachment to the basement membrane and their differing height of nuclei give it a characteristic pseudostratified appearance (14, 18, 19).

Basal cells are epithelial progenitor cells that are characterized by the expression of cytokeratins 5 and 13, and Tumour Protein 63 (TP63) that is necessary for their differentiation and formation of normal epithelium (17, 20). They are relatively undifferentiated and are anchored to the underlying basement membrane by hemidesmosomes. Basal cells have a capacity to migrate into areas of injury and differentiate into other cell types, which facilitate damage repair (17, 21).

Ciliated epithelial cells are the predominant cell type in the airway epithelium. They have characteristic projections on the apical surface (cilia) that provide forward and back-strokes in a coordinated manner to move mucus, and pathogens trapped within it, from the lower airways (22). The membrane of each cilium surrounds an axoneme which has a repeating ‘9 + 2’ structure: nine microtubule doublets which surround two central microtubules. These microtubules move relative to one another through force generated by dynein arms to move cilia in a coordinated manner (23). Ciliated epithelial cell differentiation occurs in the absence of Notch signalling. Without Notch suppression, Geminin coiled-coil containing nuclear protein (GMNC) induces Multiciliate Differentiation And DNA Synthesis Associated Cell Cycle Protein (MCIDAS), which then activates Forkhead box protein J1 (FOXJ1) (24). FOXJ1 is the primary transcriptional regulator of cilia formation, regulating the docking of basal bodies to the apical surface, the elongation of cilia and the generation of motile components (25).

The secretory cells in the epithelium are comprised of goblet cells and mucus-producing cells on the surface of submucosal glands. These cells produce mucus comprised of heavily glycosylated gel-forming mucins, which are highly water absorptive allowing them to trap and expel foreign pathogens. MUC5AC and MUC5B are the most expressed mucins, with MUC5AC predominantly expressed in goblet cells and MUC5B in submucosal glands (26, 27). Goblet cells facilitate constitutive mucus secretion while submucosal glands, controlled by autonomic innervation, can rapidly increase mucus production in response to stimulation. Notch signalling promotes goblet cell hyperplasia by supressing TP63 and GMNC and activating Sam-pointed domain Ets-like factor (SPDEF), a driver of goblet cell differentiation (24).

Club cells are found in the small airways and their gene expression is closely related to that of basal cells (28). They provide an immunomodulatory role, secreting club cell secretory protein (CCSP) which has antiprotease and anti-inflammatory activity (29, 30). In addition to their host defence and anti-inflammatory role, they have a progenitor role in the small airways with the capacity to differentiate into ciliated, secretory or alveolar cells (31, 32).

## 3 The basement membrane and cell-cell connections

The airway epithelium is a pseudo-stratified layer with clear apical and basolateral aspects that play a role in intercellular signalling (14, 15, 18). Epithelial cells sit on a basement membrane providing support and enabling communication with the underlying lamina propria. The basement membrane is an acellular structure formed of a basal lamina on top of the reticular basement membrane with proteins binding the two. The basal lamina is formed of collagen IV, laminin and proteoglycans synthesized by epithelial cells. The reticular basement membrane is formed of collagen I, III, IV and tenascin which are produced by fibroblasts in the underlying lamina propria. This functional and homeostatic complex is formed from tightly interwoven barrier and junctional complexes anchored to the basement membrane (1, 33). This binding and anchoring formed through integrin binding and hemidesmosomes stabilises the basal epithelial cell adhesion to the basement membrane (1, 33).

**Figure 1:**
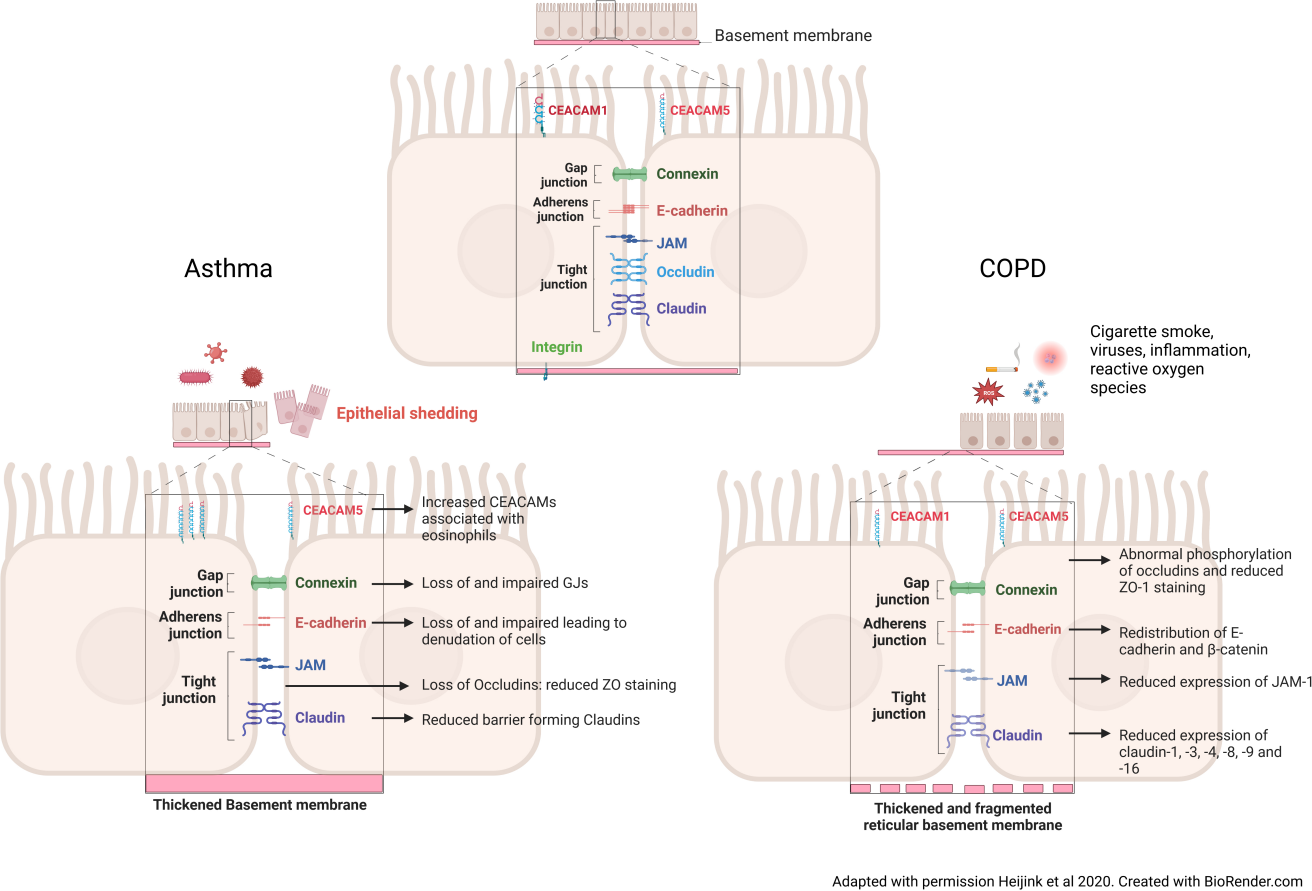
Changes to the intercellular junctions in asthma and COPD. In the asthmatic airway, after exposure to pathogens, inflammatory cytokines and the influx of inflammatory cells cause an increase in Carcinoembryonic Antigen-Related Cell Adhesion Molecule 5 (CEACAM5) expression on the apical of surface of the cells. These cytokines and inflammatory cells, also cause a loss of vital gap junction, adherens junction and of barrier forming tight junction proteins. A thickened basement membrane is a feature seen in the asthmatic airway, meaning that epithelial cell attachment to the membrane is not altered, but the cell-cell contact is reduced. Taken together this induces epithelial shedding and denudation, reduced barrier integrity and increased permeability in asthma. In the COPD airway, continued cigarette smoke exposure causes a redistribution of adherens junction proteins, namely E-cadherin and *β*-catenin, reduced tight junction protein expression through abnormal phosphorylation. Unlike the asthmatic airway, the basement membrane is fragmented, causing reduced binding of the cells to the basement membrane.

Adherens and tight junctions are complexes that regulate the airway epithelium’s permeability and maintain its barrier function against foreign antigens that are not cleared by the muco-ciliary system (**Figure 1**). The most apical of these complexes are the Tight Junctions (TJs), formed of multiple protein strands that maintain close opposition of adjacent cell membranes (1, 33). Tight junction proteins include the Zona occludin molecules (ZO-1, ZO-2, ZO-3), occludin, junctional adhesion molecules (JAMs) and claudins. These proteins maintain barrier function by binding to actin fibres and binding to each other in the cytoplasm (1, 33). Further functions of tight junctions include influencing cell morphology, cell proliferation and differentiation. The distribution and amount of specific pore-forming and barrier-forming claudins, play a role in determining the stability of the barrier (33).

Adherens junctions form another junctional layer, which consists of the transmembrane protein E-cadherin bound to cadherin-catenin complexes in the cytoplasm of adjacent cells (15, 18, 34). They play an essential role in cell-cell adhesion, differentiation, proliferation and maturation of adjacent cells (33, 34). Desmosomes are junction complexes, found near adherens junctions, which interact with the cell’s intermediate filament cytoskeleton and maintain cell mechanical integrity and polarity (1, 15, 35). Finally, the gap junctions enable macromolecules and metabolite movement between cells and, through connexins, aid in the co-ordination of the epithelial ciliated cell beat frequency (18).

Despite forming a tight barrier, basement membrane pores are important for enabling cells to move between the epithelium and lamina propria and in reverse. In epithelial-mesenchymal transition (EMT), epithelial cells lose epithelial markers (cytokeratins and E-cadherin), migrate to the lamina propria and gain mesenchymal markers (vimentin, N-cadherin, S100A4, MMP-9 and α-smooth muscle actin). These cells synthesize extracellular matrix (ECM) to provide a framework for basal cells to infiltrate and differentiate to replace damaged epithelium.

## 4 Exposures to the epithelium, the acute and chronic inflammatory response

Apart from its important role as a barrier, the airway epithelium is also involved in the regulation of the innate immune response. Airway epithelial cells have pattern-recognition receptors that recognize inhaled irritants, such as allergens and pathogens, triggering the release of cytokines, chemokines and alarmins that regulate dendritic cell, T and B cell function and trigger an acute inflammatory response. In susceptible individuals, such as patients with asthma and COPD, prolonged activation of the epithelium by inhaled irritants leads to a chronic and exaggerated immune activation. This abnormal response leads to the production of high levels of cytokines, chemokines, growth factors, proteases and ROS, which exacerbate tissue injury and inflammation, and drive abnormal repair and airway remodelling (13, 16, 36, 37).

### 4.1 Epithelium-immune cell crosstalk

The interaction of immune and epithelial cells is necessary to maintain tissue homeostasis (1). The immune response is dependent upon the co-ordinated response and activation of pattern recognition receptors (PRRs) and damage-associated molecular patterns (DAMPs) that cause immune cell activation and recruitment (1, 2). In the healthy airway, there is an effective activation and resolution of the immune cell associated repair mechanisms that are induced by and in response to epithelial injury (1). The interaction of immune cells and the airway epithelium means there is a continuous influence of the epithelial cells on the immune cells and vice versa. As discussed previously, the airway epithelium is the first line defence against any foreign particles which are recognised by the PRRs and DAMPs. The main mechanism of defence activated by the PRRs, and DAMPs are epithelial alarmin release. Epithelial alarmins including IL-25, IL-33, TSLP and HMGB and chemokine release including CCL17, CCL-11 and CCL22, activates and recruits immune cells, both directly and indirectly (1–3).

## 5 Airway epithelial dysfunction in asthma

Asthma is a chronic inflammatory disease, characterized by a heightened, continued, and chronic immune response. Patients with asthma typically have a history of variable respiratory symptoms, with reversible expiratory airflow limitation that may be associated with airway hyperresponsiveness (38). In two thirds of cases, it is linked to a history of atopy but can also be associated with occupational and other exposures (39).

Cough and mucus production are common symptoms of asthma that are correlated with worse outcomes. Post-mortem studies have shown mucus plugging to be the primary cause of death in asthma (40). Mucus hypersecretion is associated with goblet cell hyperplasia and submucosal gland hypertrophy in the epithelium (41). Recent studies, using CT and MRI imaging, have shown an association between increased mucus and features of high Th2 inflammation, ventilation mismatch and lower FEV_1_ in patients with asthma (42–44). There has been a great expansion in the development of monoclonal antibody treatments for asthma that target mechanisms including the changes in the epithelium that contribute to mucus hypersecretion (45).

In asthma, the airway epithelium shows structural and functional abnormalities, which are possibly a result of chronic injury and abnormal repair. Epithelial injury, whether due to allergen, pollution, viral or bacterial insult, leads to several changes in the response and integrity of the epithelium, which is accompanied by immune cell recruitment, inflammation, and epithelial shedding and remodelling. In the normal healthy non-asthmatic airway this process resolves. However, the asthmatic airway epithelium shows exaggerated release of cytokines/chemokines, such as IL-6, CXCL8, and alarmins, including IL-25, TSLP, IL-33, in response to injury. These mediators activate and recruit immune cells such as eosinophils, Th2 cells, macrophages and neutrophils. Macrophages and neutrophils produce proteases and ROS, whilst eosinophils release cationic proteins, which further damage the epithelial layer, and contribute to symptom exacerbations (13, 15, 16, 37, 46, 47). Activated epithelial cells and innate immune cells also release pro-remodelling factors, such as epidermal growth factor receptor (EGFR) ligands, and transforming growth factor (TGF)-*β*, which trigger goblet cell hyperplasia, EMT, fibroblast activation, and airway smooth muscle cell proliferation and hypertrophy (1, 2).

### 5.1 Epithelial structural changes in asthma

**Figure 2:**
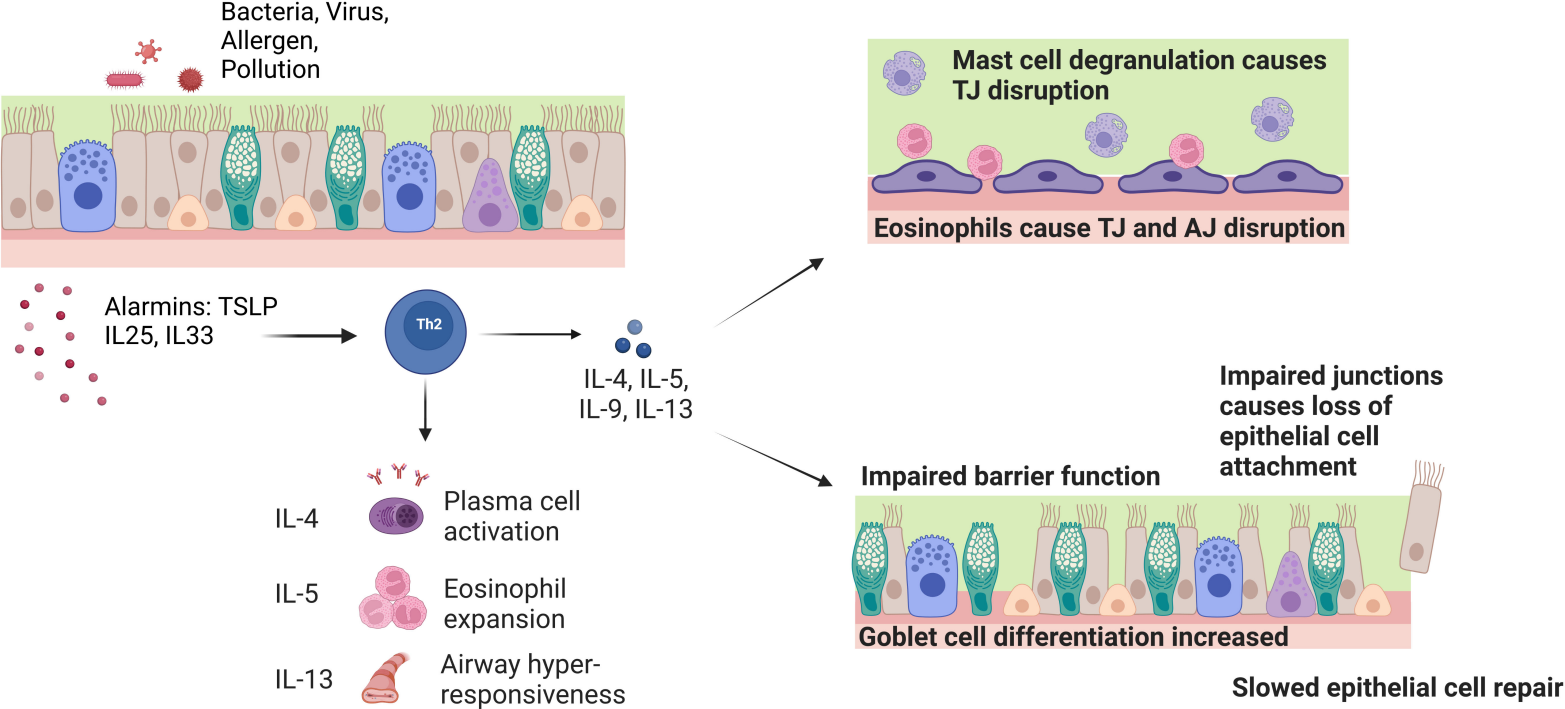
Effect of chronic inflammatory cells and cytokines on barrier function and integrity in asthma. Exposure of the airway to pathogens causes the release of epithelial cell alarmins such as TSLP, IL-25 and IL-33. The alarmins activate Th2 inflammatory cells that release the IL-4, IL-5 and IL-13 cytokines. These cytokines play a role in driving the symptoms seen in asthma such as plasma cell activation, eosinophil expansion and airway hyper-responsiveness. On a cellular level, the IL-5 induced eosinophil expansion interacts with the epithelial barrier and causes tight junction (TJ) disruption through reduction of barrier forming claudins and mast cell degranulation. IL-4 and IL-13 inhibit the expression of TJ and adherens junction (AJ) genes E-cadherin, occludin and ZO-1. These cytokines also induce goblet cell hyperplasia and defective basal cell repair causes impaired barrier function and loss of ciliated epithelial cells.

Asthmatic airways show increased numbers of basal cells with an impaired proliferation and differentiation capacity, goblet cell hyperplasia and an imbalance in the production of mucins, with increased MUC5AC and reduced MUC5B (**Figure 2**) (15, 17, 18, 22, 48–50). These two mucins have different roles within the airways: MUC5B functions in normal mucus mucociliary clearance whereas MUC5AC exacerbates airway hyperresponsiveness and mucus plugging (51). Endobronchial biopsies from patients with asthma, imaged with scanning electron microscopy, also show reduced ciliation and increased areas of squamous epithelium (52). Functionally, ciliated cells sampled from patients with severe asthma show dyskinetic movement, slower ciliary beat frequency and microtubule defects (53). Club cell markers (CCSP) are also reduced in bronchial alveolar lavage fluid taken from patients with asthma (54). CCSP has also been shown to increase following allergen challenge in patients with allergic asthma, suggesting acute leakage due to club cell damage in the small airway epithelium (55).

Abnormal cell differentiation may contribute to these abnormalities. EGFR, which is increased in the asthmatic epithelium, is a driver of abnormal cell differentiation and epithelial dysfunction (56–58). Furthermore, IL-13, elevated in allergic asthma, inhibits ciliated cell differentiation and causes reduced ciliary beat frequency and abnormal distribution of basal bodies, whilst enhancing goblet cell differentiation (17, 49). Specifically, IL-13 upregulates SPDEF and MUC5AC expression, whilst downregulating FOXJ1 and other cilia-related transcription factors via activation of JAK/STAT and Notch signalling and changes in histone methylation (59–61).

### 5.2 Epithelial integrity

In asthma, epithelial defects in the adherens, tight and gap junctions, as well as altered permeability and integrity, are present from the nose through to the lower airways (**Figure 1**). Junctional complex disruption is thought to occur through hyperphosphorylation and decreased expression of complex genes and proteins in asthmatic patients because of deeper penetration and increased immune response to particles (1, 17, 62). The disrupted and leaky barrier in asthma is well-documented and a positive feedback loop of IL-4 and IL-13 contributes to this phenotype seen in patients with asthma. Reduced expression of the barrier forming claudins -1, -3, -4 and -18 is associated with increased IL-13 production in asthma, from mild-moderate to severe asthma (14, 15, 33). Reduced ZO-1 and E-cadherin expression are evident in asthma where they cause denudation and detachment of ciliated epithelial cells (1, 14, 15, 62).

Ciliated epithelial cell detachment is paired with loss of cell-cell contacts and reduced integrity (12, 14, 15, 63, 64). This leads to pro-inflammatory cytokine release and recruitment of immune cells, which induce apoptosis and further damage the epithelial layer (12, 14, 15, 63, 64). Apoptosis and autophagy occur in the non-asthmatic airway epithelium and are crucial for airway epithelial homeostasis (21, 64). These mechanisms regulate the number of proliferating epithelial cells and remove damaged cells without the induction of a chronic inflammatory response (21, 64). However, in asthma there is increased apoptosis of ciliated epithelial cells leading to epithelial shedding and barrier dysfunction, which increases with disease severity.

Environmental pathogenic factors, including allergens, air pollutants and respiratory infections, and the associated immune response, cause airway epithelial injury (**Figure 2**). Allergen proteases, such as Der p1 in house dust mites, induce inflammation and disrupt the epithelial barrier either directly by cleaving tight junction proteins, or indirectly by activating protease activated receptor (PAR)-2 (14, 65, 66). PAR2-dependent signalling promotes epithelial junction disassembly through ZO-1 and occludin degradation, and EGFR-dependent redistribution of E-cadherin (67). Furthermore, ATP released from the injured epithelium may also promote loss of epithelial integrity through activation of purinergic receptors (68). EGFR activation by allergens also promotes EMT in airway epithelial cells (69). Allergens promote oxidative stress, which triggers epithelial cell death and inflammation through oxidative damage of DNA and mitochondria (70–74). Mitochondrial dysfunction has been shown to cause ROS-mediated disruption of barrier function in intestinal epithelium, whilst reduced E-cadherin expression and loss of barrier function are associated with attenuated mitochondrial biogenesis in airway epithelial cells (75, 76). Viral infections and inhaled pollutants can also cause epithelial injury by inducing cell death and disruption of tight junctions (77–80).

The asthmatic airway immune response also leads to loss of epithelial integrity. Mast cell degranulation, IL-5 and direct contact with eosinophils, leads to disruption of tight junctions and increased permeability (17, 37, 81). Furthermore, the T2 cytokines IL-4 and IL-13 inhibit the expression of the key tight and adherens junction genes ZO-1, E-cadherin and occludin, and drive goblet cell differentiation from basal cells (**Figure 2**) (13, 82).

Susceptibility to epithelial injury by environmental irritants, and abnormal repair mechanisms, are key factors in asthma pathogenesis. House dust mites, extracts from the allergenic fungus *Alternaria Alternata* and cigarette smoke were shown to induce greater barrier dysfunction in air-liquid-interface (ALI) cultures of asthmatic airway epithelial cells compared to those of healthy subjects (68, 83, 84). Inadequate antioxidant and antiviral mechanisms in asthmatic airways may also confer epithelial cells more susceptibility to oxidative and viral-induced damage (72, 85, 86). Epithelial cells from patients with asthma are also abnormally slow at repairing injury in *in vitro* models. This is possibly caused by asynchronous mitosis of epithelial cells and is associated with increased production of TGF-β which drives EMT and ECM production (87–89).

Taken together, the data suggests that there is a vicious cycle of epithelial cell injury and immune activation, which in conjunction with increased susceptibility and abnormal repair mechanisms promote an abnormal airway epithelium in asthma (13, 16, 37).

### 5.3 Epithelium-immune cell crosstalk in asthma

In the asthmatic airway, alarmin and chemokine release induced cytokine activation is a well-recognised disease driving mechanism. The release of alarmins primes the Th2 CD4 T-cell response through maturing dendritic cells (DCs) via CCL17 (42). DCs in asthma mount an uncontrolled Th2 response and acts to enhance ILC2, basophil and mast cell functionality (90). The epithelium response to injury enhances the Th2 release of pro-inflammatory cytokines such as IL-4 from mast cells and basophils increasing levels of IgE, IL-5 for the activation and maturation of eosinophils in the bone marrow and IL-13 that induces hyper-responsive smooth muscle contraction, goblet cell hyperplasia and the activation of macrophages (90).

The interaction between the epithelium and immune cells can influence many different parts of the airway through alarmin release; in the innate immune system, DCs cause increased activation of Th2 cells, basophils have increased histamine degranulation and ILC2 cells release increased pro-inflammatory cytokines (17). In the epithelial and endothelial cells, a feedback loop is induced, the epithelium becomes leakier due to cleaved junctions, smooth muscle cells become hyper-responsive and fibroblasts release increase CCL5, GM-CSF and CXCL8 (17).

## 6 Airway epithelial dysfunction in COPD

COPD is a respiratory disease characterised by chronic irreversible airflow obstruction, which manifests as shortness of breath, cough and sputum production. Breathlessness typically increases over time and is interspersed by periods of worsening respiratory symptoms known as exacerbations, which accelerate lung function decline.

**Figure 3:**
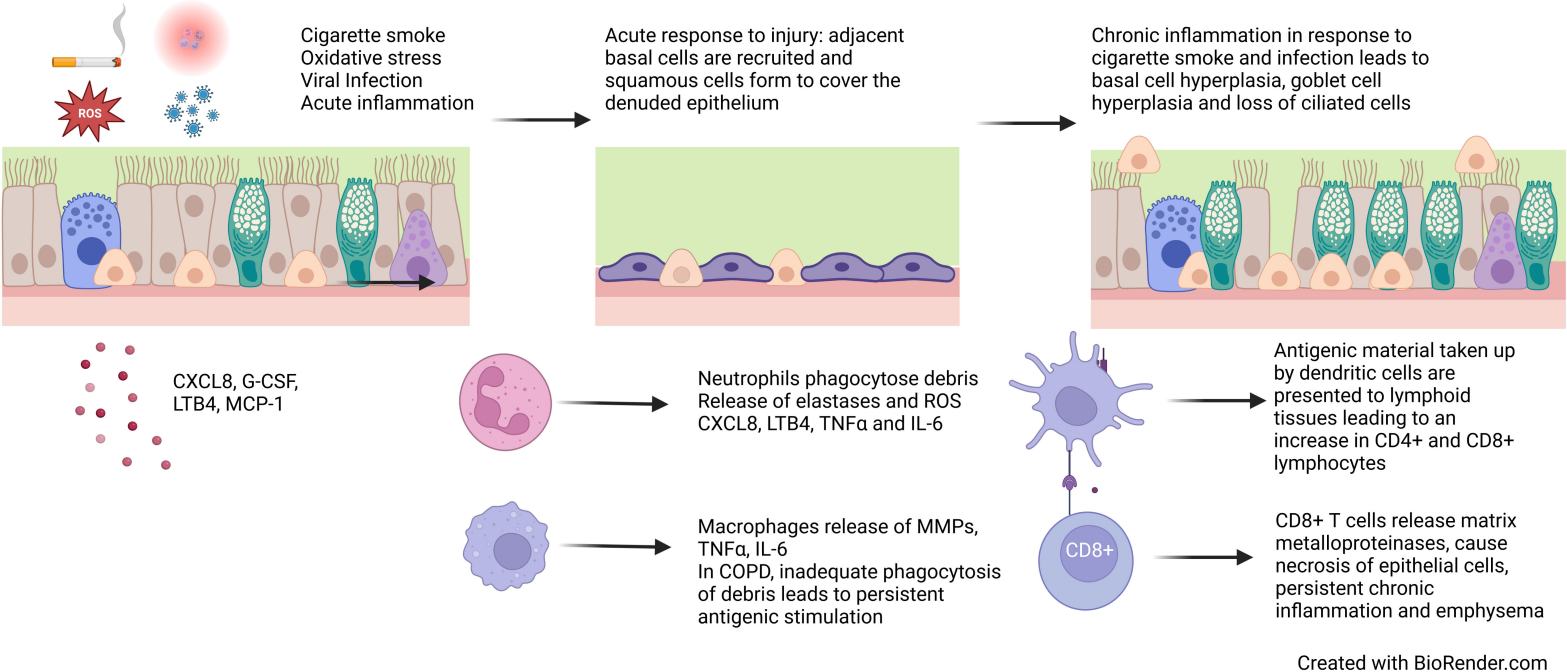
Epithelial changes in COPD. Cigarette smoke, reactive oxygen species, viral infection and acute inflammation leads to loss of ciliated epithelial cells and squamous metaplasia. Persistent exposures and chronic inflammation lead to basal cell hyperplasia, goblet cell hyperplasia and reduced ciliation.

Chronic bronchitis is a clinical phenotype of COPD which involves “chronic cough and sputum production for three months a year for two consecutive years” (91). Patients with chronic bronchitis have poorer quality of life, more frequent exacerbations, faster lung function decline and increased mortality (54, 91–93). COPD and chronic bronchitis are characterised by epithelial changes that contribute to chronic mucus secretion (**Figure 3**). A reduction in ciliated cells and an increase in secretory cells lead to more mucus, which is more difficult to clear and contributes to symptoms, repeated infections and faster lung function decline.

### 6.1 Changes to the epithelial structure in COPD

There is evidence of increased numbers of basal cells in the airways of patients with COPD, which however show compromised capacity to regenerate and differentiate (94). EGFR is expressed in basal cells and can be activated by EGF produced by ciliated cells in response cigarette smoke and inflammatory mediators (5, 6). EGFR is increased in smokers and is associated with increased mucous and squamous metaplasia via Notch signalling (3).

Samples taken at bronchoscopy from people who smoke have shown a loss of cilia, shorter cilia and ultrastructural defects in the axoneme (8, 9, 90, 95–97). Gene expression of dynein arms (DNAH5, DNAH9 and DNAH11) that drive cilia beating, and intraflagellar transport proteins (IFT43, IFT 57, IFT144 and IFT172), which determine cilia length and development, have been shown to be reduced in smokers. Within COPD, similar features of loss of cilia, shorter cilia and slower cilia are seen when compared to healthy controls and healthy smokers (8, 98). Although inhaled therapies for COPD, β_2_-agonists and muscarinic antagonists, can increase ciliary movement these effects are blunted by high levels of inflammation (99, 100).

Goblet cells are increased in smokers and patients with COPD, which is thought to lead to an increase in baseline mucus production (7). Although submucosal gland density does not appear to be increased in COPD, gland inflammation is increased and may contribute to increased mucus release in response to infectious and non-infectious triggers (10). The primary mucins, MUC5AC and MUC5B are both increased in COPD (101–104). Furthermore, club cells are reduced in the small airway epithelium of patients with COPD (105). CCSP is reduced in smokers and patients with COPD and has been shown to correlate with FEV_1_ (106, 107).

### 6.2 Epithelial integrity and permeability

In COPD, there is a known and recognised defect in the epithelial barrier permeability and integrity in response to stressors (**Figure 1**). In epithelial cells from patients with COPD, reduced expression of occludins, claudins and JAM-1 is seen. Oxidative stress leads to abnormal phosphorylation of occludin and redistribution of occludins, ZO-1, E-cadherin and β-catenin throughout the junction (79). Increased permeability of the epithelium is seen in response to cigarette smoke and associated chronic inflammation, with a reduction in staining of ZO-1 (108, 109). Many viruses including rhinovirus also reduce ZO-1, and further lead to reduced epithelial resistance and increased permeability in ALI cultures (110), Epithelial cells exposed to TNFα and IFN-γ also show reduced ZO-1 and JAM staining and attenuated barrier function (111). This process of inflammation-induced loss of tight and adherens junctions predisposes the epithelium to further infection and exacerbates the persistent chronic inflammatory state seen in COPD.

The reticular basement membrane in COPD is thicker than in healthy controls but as not thick as seen in patients with asthma (112, 113). However, fragmented reticular basement membrane is seen in COPD and healthy smokers (114). In COPD, however, repeated trauma leads to persistent EMT activation through TGF-β activation and further basement membrane fragmentation (115, 116). EMT markers have been shown be increased in COPD and to correlate with the severity of airflow obstruction (117, 118).

### 6.3 Chronic inflammation in COPD

Chronic inflammation is a key factor in COPD pathogenesis resulting from repeated insults and oxidative stress, which cause cell death, senescence and abnormal cell differentiation and function. While there are many risk factors, cigarette smoke is the most common in COPD. Cigarette smoke contains reactive oxygen species (ROS) which can be directly cytotoxic to epithelial cells and trigger an acute inflammatory response, which is further exacerbated by bacterial and viral infections. Epithelial cells release pro-inflammatory cytokines CXCL8, G-CSF, LTB4 and MCP-1 resulting in the recruitment of monocytes, macrophages and neutrophils which in turn release IL-6 and TNFα (4). The inflammatory response and oxidative stress lead to necrosis and apoptosis of epithelial cells. In response to this trauma, cells adjacent to the area de-differentiate to cover the denuded area, forming a flattened area of squamous epithelium to protect against future damage (119). In health, if toxic exposure to the epithelium ceases, basal cells and CD45- stem cells differentiate for form a pseudostratified epithelium (120, 121). However, in COPD, persistent exposures and chronic inflammation prevent the formation of structurally and functionally normal epithelium (**Figure 3**).

In the COPD epithelium, the chronic inflammatory process occurs due to ongoing oxidative stress (usually from cigarette smoke), persistent activation of inflammatory cells through cell damage and impaired processes to fight infection. Toll-like receptors on epithelial cells are directly stimulated by cigarette smoke activating a cascade of inflammatory mediators, further tissue damage and release of antigenic material (122, 123). Antigens are also taken up by dendritic cells (which are increased in the COPD epithelium) and are presented to CD8+ T cells (124, 125). CD8+ T cells are increased in COPD and numbers inversely correlated with FEV_1_ (126). These cells are cytotoxic to epithelial cells and release proteolytic enzymes including matrix metalloproteases (MMPs) which can drive emphysema, a clinical feature of COPD. Phagocytes, in particular neutrophils and macrophages, are altered in COPD. Neutrophils and macrophages engulf microbes and apoptotic cells. Neutrophils and macrophages are increased in the epithelium and are associated with chronic bronchitis, more severe airflow obstruction and faster lung function decline (127–129). Despite being increased in number, they show impaired phagocytic ability which predispose to recurrent infection (130–132). Neutrophils and macrophages also release reactive oxygen species, pro-inflammatory cytokines, growth factors and MMPs, which propagate chronic inflammation, emphysema, and airway remodelling processes, such as EMT, fibroblast activation and airway smooth muscle proliferation (1) (**Figure 3**).

### 6.4 Epithelium-immune cell crosstalk in COPD

The continued interaction of the immune cells and epithelium (39, 133) in COPD is facilitated by the proximity of DCs to the airway epithelial cells. In COPD, DCs have been identified as upregulating the expression of chemokines CXCL10, CCL-20 and CCL-10 that cause the activation of T-cells, predominantly a Th1-pro inflammatory profile (39, 133). Epithelial alarmin release of IL-33 has also been identified as being upregulated in COPD; this has been shown to cause an increase in neutrophil and macrophage infiltration and activation. This drives a positive feedback loop on both epithelial cells and immune cell upregulation of IL-6 and CXCL8 release (39). The up-regulation of HGMB1 on epithelial cells in COPD stimulates increased IL-1b production and release from activated macrophages (39, 133).

### 6.5 The role of oxidative stress in epithelial dysfunction

ROS, from cigarette smoke or the inflammatory response, drive cell death, senescence, abnormal cell-cell connections and abnormal remodelling (134, 135). Epithelial cells have antioxidant mechanisms to mitigate the ill effects of ROS but these are impaired in COPD which renders these cells more susceptible to oxidative damage (72, 136–138). Epithelial cells in COPD, have a dysregulated redox state which promotes airway inflammation and remodelling by regulating the activity of ROS-dependent kinases, phosphatases, transcription factors and epigenetic effectors, and by drivingTGF- *β* expression and EGFR activation (139–141).

DNA damage occurs in response to oxidative stress and is associated with nucleotide base oxidation, and single and double-strand DNA breaks in COPD (142, 143). In addition to the increased insults, DNA repair processes are defective in patients with COPD (143, 144). These along with telomere damage, as a result of oxidative stress, lead to upregulation of the cell cycle inhibitor p16^INK4^ and mammalian target of rapamycin (mTOR)-mediated signalling to trigger premature senescence in COPD airway epithelial cells (145–149). Although acute senescence may play a role in damage repair, prolonged senescence may lead to impaired epithelial regeneration and damage repair by limiting the proliferative capacity of progenitor cells. Furthermore, although in cell cycle arrest, senescent cells remain metabolically active and secrete a multitude of proteases, cytokines, chemokines and growth factors, such as TGF-*β* (148). Therefore, accumulation of senescent cells may exacerbate lung inflammation and epithelial injury, and drive emphysema and fibrosis (150–155).

Mitochondria are major sites of oxidative damage in COPD epithelial cells. Cigarette smoke alters mitochondrial morphology and respiration in normal bronchial epithelial cells, whilst bronchial epithelial cells from COPD patients show abnormal mitochondrial morphology, possibly as a result of prolonged exposure to oxidative stress (156, 157). Mitochondrial dysfunction leads to increased production of ROS, which in turn cause further mitochondrial damage. This creates a vicious cycle of oxidative stress and mitochondrial dysfunction that drives pathology. Specifically, mitochondrial ROS promote apoptosis, senescence and inflammatory mediator release in airway epithelial cells, as well as impaired cellular function manifesting as reduced mucociliary clearance (158–161). Mitochondrial dysfunction also triggers ROS-dependent activation of necroptosis, a lytic form of cell death, that is accompanied with the release of immunogenic damage-associated molecular patterns (DAMPs). DAMPs, which include mitochondrial molecules such as ATP and cardiolipin, promote further inflammatory mediator release and result in increased MUC5AC expression in the airway epithelium (162–164).

Autophagy is a cellular process that is pivotal in the adaptation to oxidative stress. In this process, damaged molecules and organelles are engulfed into double-membrane bound vesicles called autophagosomes and fuse with lysosomes leading to their degradation. Reduced autophagic activity in COPD lungs, resulting from prolonged oxidative stress and ageing, may lead to insufficient removal of damaged cellular components and premature airway epithelial cell senescence (165–168). Specifically, impaired autophagic removal of mitochondria (mitophagy) has been reported in the airway epithelium of COPD patients, where it leads to accumulation of damaged mitochondria and epithelial cell senescence through ROS-mediated DNA damage (161, 169–171). However, other studies show excessive autophagic activity in COPD, leading to airway epithelial cell apoptosis, necroptosis and inflammatory mediator release (172–174). Cigarette smoke-induced autophagy has also been shown to cause degradation of ciliary proteins, and to promotes MUC5AC expression through activation of c-Jun N-terminal kinase (JNK) and activating protein (AP)-1 (175, 176). Therefore, dysregulated autophagy leads to the abnormal adaptation of airway epithelial cells to injury and contributes to airway remodelling.

### 6.6 Epigenetic and genetic determinants

Predisposing factors combined with exposures both in early life and adulthood likely contribute to epithelial pathogenesis seen in COPD. Gene polymorphisms, and epigenetic changes including DNA methylation, histone modifications and non-coding RNAs, may play a role in susceptibility and abnormal repair following epithelial injury in COPD (152, 177).

DNA methylation at gene promoters, a modification associated with reduced gene expression, is altered by cigarette smoking in airway epithelial cells (178–180). In ALI cultures derived from COPD bronchial epithelial cells, DNA hypomethylation at the SPDEF gene promoter leads to increased MUC5AC expression (178). In cigarette smoke extract-exposed COPD airway epithelial cells, gene promoter hypomethylation triggers increased expression of the aryl hydrocarbon receptor repressor (AHRR), an inhibitor of the cytoprotective transcription factor aryl hydrocarbon receptor (AHR). This leads to inhibition of AHR expression and increases susceptibility to CS-induced epithelial cell apoptosis and necroptosis (180). Conversely, reduced expression of the antioxidant transcription factor nuclear factor E2-related factor 2 (Nrf2) due to DNA hypermethylation in COPD, may increase the susceptibility of bronchial epithelial cells to oxidative stress-dependent cell death (181).

Acetylation of lysine residues on histones, which is catalysed by histone acetyltransferases and removed by histone deacetylases, is usually associated with open chromatin configuration and active gene transcription. Histone methylation on lysine and arginine residues, regulated by histone methyltransferases and demethylases, can activate or inhibit gene expression depending on the site and number of methyl groups added (182). An imbalance between histone acetylation and deacetylation, favouring acetylation, has also been observed in COPD lungs and has been associated with increased inflammatory gene expression (167, 179). Reduced expression of histone acetyltransferase binding to ORC1 (HBO1) in COPD leads to downregulation of anti-apoptotic protein Bcl-2, sensitising airway epithelial cells to oxidative stress-mediated apoptosis (183). Furthermore, reduced expression of the arginine methyltransferase coactivator-associated arginine methyltransferase 1 (CARM1) in COPD bronchial epithelial cells is associated with increased senescence, and impaired club cell regeneration and epithelial repair, possibly through altered histone methylation of cell cycle and differentiation genes (184, 185).

miRNAs are small non-coding RNAs that regulate gene expression through the induction of mRNA degradation and inhibition of translation. A number of miRNAs have been shown to be abnormally expressed in COPD airways where they are involved in airway epithelial inflammation, mucous hypersecretion, EMT and cellular senescence (152, 186–188).

Some of the gene polymorphisms identified by genome-wide association studies to be associated with COPD may increase susceptibility to epithelial injury. A gene variant of FAM13A, a protein expressed mainly in club cells and alveolar type 2 cells, is associated increased gene expression and reduced lung function in COPD. FAM13A is an inhibitor of Wnt signalling, a regulator of epithelial differentiation, therefore its upregulation may contribute to impaired club cell differentiation in COPD airways (189). Furthermore, FAM13A promotes fatty acid oxidation and mitochondrial respiration by driving the expression of carnitine palmitoyltransferase 1A (CPT1A), contributing to ROS-mediated bronchial epithelial apoptosis (190). However, FAM13A inhibits TGF-*β-*mediated EMT in airway epithelial cells, indicating it may also have protective effects against airway remodelling (191). Variants of the iron-regulatory protein 2 (IRP2) have also been associated with COPD susceptibility. IRP2 is found in airway epithelial cells, predominantly at the cilial surface. Increased IRP2 expression in COPD causes mitochondrial dysfunction due to iron overload, leading to increased airway epithelial cell death, inflammation and impaired mucociliary clearance (192, 193).

## 7 Discussion

The epithelium protects the airways against pathogens and inhaled irritants. It achieves this by acting as a physical barrier preventing them from entering into the submucosa, by trapping them in mucous and expelling them through ciliary clearance, and by orchestrating their clearance by the immune system. In asthma and COPD, inherent defects in protective and repair mechanisms in conjunction with repetitive injury, lead to significant structural and functional abnormalities in the epithelium. Specifically, mucous overproduction and ciliary dysfunction in COPD leads to defective clearance and increased entry of pathogens into the airway wall, triggering an immune response. In asthma, the disruption of tight junctions and adherens junctions between epithelial cells, epithelial shedding and deeper penetration of pathogens, again triggers a constant feedback of immune cell response. In both diseases, this leads to a continuous cycle of injury and abnormal repair that drives chronic airway inflammation and remodelling. Targeting the mechanisms of damage susceptibility and abnormal repair in the epithelium may lead to new and more effective therapies for asthma and COPD. Future work studying the effects of biologic therapies in asthma on barrier function needs to be explored. Studying how removing certain key cytokines such as IL-5, IL-4 and alarmins TSLP can alter the epithelial response to injury and how this could potentially change the epithelial-immune cell interaction. Studying key proteins and pathways that are altered in asthma and COPD epithelial barrier, such as Notch signalling, Wnt signalling, and gap junctions should be the future direction of studying epithelial barrier integrity alterations.

## Author contributions

KR, CM, and JT wrote the manuscript. KR, CM, JT, KC, and PB conceived and finalised the manuscript.
